# A Novel Competing Endogenous RNA Network Associated With the Pathogenesis of Graves’ Ophthalmopathy

**DOI:** 10.3389/fgene.2021.795546

**Published:** 2021-12-15

**Authors:** Zifan Yue, Pei Mou, Sainan Chen, Fei Tong, Ruili Wei

**Affiliations:** Department of Ophthalmology, Changzheng Hospital of Naval Medicine University, Shanghai, China

**Keywords:** Graves’ ophthalmopathy, microRNA, long noncoding RNA, circular RNA, RNA sequencing

## Abstract

**Background:** Growing evidence has recently revealed the characteristics of long noncoding (lncRNA)/circular RNA (circRNA)-microRNA (miRNA)-mRNA networks in numerous human diseases. However, a scientific lncRNA/circRNA-miRNA-mRNA network related to Graves’ ophthalmopathy (GO) remains lacking.

**Materials and methods:** The expression levels of RNAs in GO patients were measured through high-throughput sequencing technology, and the results were proven by quantitative real-time PCR (qPCR). We constructed a protein-protein interaction (PPI) network using the Search Tool for the Retrieval of Interacting Genes (STRING) database and identified hub genes by the Cytoscape plug-in CytoHubba. Then, the miRNAs related to differentially expressed lncRNAs/circRNAs and mRNAs were predicted through seed sequence matching analysis. Correlation coefficient analysis was performed on the interesting RNAs to construct a novel competing endogenous RNA (ceRNA) network.

**Results:** In total, 361 mRNAs, 355 circRNAs, and 242 lncRNAs were differentially expressed in GO patients compared with control patients, 166 pairs were identified, and ceRNA networks were constructed. The qPCR results showed that 4 mRNAs (THBS2, CHRM3, CXCL1, FPR2) and 2 lncRNAs (LINC01820:13, ENST00000499452) were differentially expressed between the GO patients and control patients.

**Conclusion:** An innovative lncRNA/circRNA-miRNA-mRNA ceRNA network between GO patients and control patients was constructed, and two important ceRNA pathways were identified, the LINC01820:13-hsa-miR-27b-3p-FPR2 ceRNA pathway and the ENST00000499452-hsa-miR-27a-3p-CXCL1 pathway, which probably affect the autoimmune response and inflammation in GO patients.

## Introduction

Graves’ ophthalmopathy (GO), also called thyroid-related ophthalmopathy (TAO), is an autoimmune orbital inflammatory disease that can cause inflammatory infiltration and fat formation in orbital tissues as well as thickening of the extraocular muscles (Şahlı and Gündüz, 2017). Consequently, GO patients have clinical signs such as eyelid retraction, exophthalmos, soft tissue inflammation, eye movement disorder, strabismus, and, in severe cases, even blindness (Antonelli et al., 2020). Recent studies have found that the orbital fibroblasts (OFs) of GO patients specifically express thyroid-stimulating hormone receptor (TSHR), which are recognized specifically by helper T cells for their activation, secrete cytokines and inflammatory factors, and promote adipogenesis and the production of hyaluronic acid. Connective tissue remodeling leads to different degrees of orbital fat swelling and enlargement of extraocular muscles (Smith, 2015). In addition to TSHR, OFs of GO patients express numerous insulin-like growth factor 1 receptors (IGF-1R) (Smith and Janssen, 2019). However, the precise mechanisms by which GO occurs and the related processes have not been explicitly elucidated. It is therefore necessary to clarify the potential molecular mechanisms and develop valid therapeutic targets and novel predictive biomarkers for GO.

Recently, numerous researchers have tried to further explore GO pathogenesis by comparing differentially expressed RNAs and proteins between case groups and control groups; noncoding RNAs (ncRNAs), which are transcription products of RNA but do not encode proteins, have especially been considered. NcRNAs can regulate both whole organisms and cells in various ways, such as via molecular functions (MFs) and by affecting biological processes (BPs). NcRNAs include circular RNAs (circRNAs), long noncoding RNAs (lncRNAs) and small noncoding RNAs (sncRNAs) (Matsui and Corey, 2017). MicroRNAs (miRNAs) are endogenous sncRNAs that have become a popular research topic in the field of bioinformatics in recent years. MiRNAs are between 20 and 25 nt long; can target mRNAs for posttranscriptional regulation; and participate in important BPs such as cell polarization, growth, senescence, and death (Jang et al., 2018). Moreover, recent studies have revealed that miRNAs have crucial effects on GO pathogenesis (Martínez-Hernández et al., 2018). For example, miR-146a is upregulated by inflammatory stress in OFs in GO patients and has effects on downregulating related target genes such as IL-1 receptor-related kinase (IRAK1) and TNF receptor-related factor 6 (TRAF6) to inhibit the NF-κB pathway, which causes the alleviation or termination of the immunoreaction (Jang et al., 2016). MiR-155 has indispensable effects on the development of Th17 cells in the autoimmune process, and the abovementioned Treg cells and Th17 cells have substantial effects on the pathogenesis of GO. In addition, miR-155 plays an indispensable role in the formation of fibrous tissue and mediates the TGF-β signaling pathway to drive collagen synthesis (Lu et al., 2009; Leng et al., 2011; Eissa and Artlett, 2019). Moreover, the roles of long lncRNAs and circRNAs in the pathogenesis of TAO have also been studied (Wu et al., 2020a; Wu et al., 2020b), suggesting that ncRNAs play pivotal roles in the pathogenesis of GO. LncRNAs are long-chain multifunctional RNAs whose length exceeds 200 nt. LncRNAs are structurally similar to mRNAs but do not encode proteins; lncRNAs interact with DNA, RNA and protein and have unique biological functions (Quinn and Chang, 2016). CircRNAs are RNA molecules with a closed loop structure generated from the covalent bonding of the 3′ and 5′ ends after reverse splicing. Therefore, circRNAs are not affected by RNA exonucleases and have organization, timing and structural specificity. Like miRNAs, circRNAs participate in gene transcription and posttranscriptional regulation and can also bind to RNA-like RNA-binding proteins to participate in regular biological functions (Huang et al., 2020). In recent years, a novel hypothesis about lncRNA/circRNA-miRNA-mRNA interactions has arisen, which is called the competing endogenous RNA (ceRNA) hypothesis (Salmena et al., 2011); it is hypothesized that mutual interference occurs between both coding and noncoding RNAs through miRNA response elements (MREs), forming an extensive regulatory network across the transcriptome. CircRNAs/lncRNAs have MREs that are similar to mRNAs that can competitively bind miRNAs to regulate mRNA levels. Generally, when ceRNA expression is silenced, mRNA is degraded under the action of the miRNA-mediated silencing complex (RISC). When ceRNA expression is activated, ceRNAs compete to bind to the RISC complex, reduce miRNA inhibitory function, and upregulate the expression of target genes (Karreth and Pandolfi, 2013). This hypothesis probably interprets disease processes and offers opportunities for new therapies. The ceRNA hypothesis has been confirmed in many disease fields, including cancer (Wang R. et al., 2018; Zhang H. et al., 2019), Alzheimer’s disease (Zhang Y. et al., 2019), myasthenia gravis (Wang et al., 2020), and autoimmune diseases (Li et al., 2018; Wu et al., 2019). Nevertheless, current knowledge of lncRNAs/circRNAs-miRNAs-mRNAs in human diseases is lacking, especially in GO.

In this study, the differentially expressed mRNAs and lncRNAs/circRNAs of the GO group and the normal group were identified through high-throughput sequencing technology, functional enrichment analysis of the differentially expressed genes was performed, and protein-protein interaction (PPI) analysis was used to identify the hub genes. Next, through seed sequence matching analysis, we predicted the miRNAs related to the differentially expressed lncRNAs, circRNAs and mRNAs and identified lncRNA/circRNA-miRNA- and mRNA-miRNA-related pairs. Then, the intersection of the lncRNA/circRNA-miRNA related pairs and the mRNA-miRNA related pairs was used to determine the intersecting miRNAs, and correlation coefficient analysis was performed on the intersecting lncRNAs/circRNAs and mRNAs to identify effective lncRNA/circRNA-mRNA pairs. Furthermore, gene expression values were used to establish relationships between circRNAs/lncRNAs, mRNAs, and miRNAs, to infer whether miRNAs regulate circRNAs/lncRNAs and mRNAs and to determine the relationships. Finally, we established a novel ceRNA network, and these network components may serve as therapeutic targets or promising diagnostic biomarkers for GO in recent years.

## Materials and Methods

### Ethics Approval

This research was permitted by the Changzheng Hospital Ethics Committee Affiliated with Naval Military Medical University. All the patients included in the experiment signed an informed consent form, and the implementation of the experiment complied with the Declaration of Helsinki.

### Patients and Tissue Samples

Orbital adipose/connective tissue was excised from 5 GO patients with no history of thyroid disease, inflammatory disease, autoimmune disease, or orbital disease by orbital decompression for proptosis correction. and control subjects underwent cosmetic upper and lower blepharoplasty. The 5 control groups were obtained from the tissue remains of healthy people after plastic surgery who underwent cosmetic upper and lower blepharoplasty. None of the patients received corticosteroid pulse therapy or orbital radiation therapy within 6 months before surgery.

### Microarray Sequencing and Analysis

We extracted total RNA from the paired tissues of 3 GO patients and 3 healthy individuals with an RNeasy Micro Kit (Qiagen, GmBH, Germany) and calculated the RNA integrity number (RIN) to check the RNA integrity with an Agilent Bioanalyzer 2100 (Agilent Technologies, Santa Clara, CA, US). Then, we used RNA samples to produce fluorescently labeled complementary RNA (cRNA) targets for the SBC human ceRNA array version 1.0 (4 × 180 K, designed by Shanghai Biotechnology Corporation and made by Agilent Technologies), which includes 88371 circRNA probes, 77103 lncRNA probes, and 18,853 mRNA probes, to identify the differentially expressed mRNAs, lncRNAs and circRNAs.

We amplified and labeled total RNA with a Low Input Quick Amp Labeling Kit (One Color) (Agilent Technologies, Santa Clara, CA, US). Then, the labeled cRNA was purified with an RNeasy Mini Kit (Qiagen, GmBH, Germany). We used a Gene Expression Hybridization Kit (Agilent Technologies, Santa Clara, CA, US) to hybridize each slide with 1.65 μg of Cy3-labeled cRNA in a hybridization oven (Agilent Technologies, Santa Clara, CA, US). Then, we washed the slides in staining dishes (Thermo Shandon, Waltham, MA, United States) with the Gene Expression Wash Buffer Kit (Agilent Technologies, Santa Clara, CA, United States) after 17 h of hybridization. The slides were then scanned with an Agilent Microarray.

Scanner (Agilent Technologies, Santa Clara, CA, US). The data were extracted with Feature Extraction software version 10.7 (Agilent Technologies, Santa Clara, CA, US), and the raw data were normalized by the quantile algorithm and limma packages in R.

### Differential Expression Analysis

We screened the differentially expressed genes using fold-change (FC) (in expression difference) and *t*-test (Student’s t-test) statistical methods after the original data were normalized with the limma package in R software. When we finished the differential expression analysis, a |log2FC| > 1 and *t*-test *p*-value < 0.05 were considered statistically significant.

### PPI Network and Hub Gene Identification

We used the Search Tool for the Retrieval of Interacting Genes (STRING) database (http://stringdb.org/) (Szklarczyk et al., 2019) to construct PPI networks between the differentially expressed genes. The sequences of the differentially expressed genes were input into the database, and high-resolution bitmaps were obtained. Only the interactors with a combined confidence score≥0.4 appear in the bitmap. By calculating the degree of connectivity, the hub genes in the PPI networks were identified using CytoHubba, a plugin for Cytoscape software (version 3.6.1) (Shannon et al., 2003). The PPI networks and the top 30 hub genes were visualized according to their node degree in Cytoscape software (version 3.6.1).

### Construction of the ceRNA Network

Seed sequence matching analysis by miRanda (http://www.microrna.org/microrna/) (Betel et al., 2008) was used to forecast the miRNAs related to the differentially expressed or interesting lncRNAs/circRNAs and mRNAs to determine the lncRNA/circRNA-miRNA and mRNA-miRNA pairs. We overlapped the two predicted results and then overlapped the results with the differentially expressed miRNAs verified by previous experiments for cross-validation to identify effective lncRNA/circRNA-mRNA pairs. Finally, correlation coefficient analysis was used to select the effective lncRNA/circRNA pairs. With a correlation value > 0.95, we established a lncRNA/circRNA-miRNA-mRNA network.

### Bioinformatic Analyses

The clusterProfiler package in R was used to conduct Gene Ontology functional annotation and KEGG pathway enrichment analysis of differentially expressed mRNAs from the lncRNA/circRNA-miRNA-mRNA network. First, the numbers of mRNAs associated with BPs, cellular components (CCs), and MFs were determined. Then, Gene Ontology enrichment analysis was performed by Fisher’s exact test, with a q-value ≤ 0.05 used as the threshold. Gene Ontology terms that met this condition were significantly enriched in differentially expressed genes, and the results are displayed in a scatter plot. Similarly, the number of differentially expressed genes that differed in each pathway was determined, the results of which are shown in a bar chart.

### Validation of the Expression Levels of mRNAs, lncRNAs and circRNAs

To validate the changes in mRNAs, lncRNAs and circRNAs detected via RNA sequencing (RNA-seq), the hub genes included in the lncRNA/circRNA-miRNA-mRNA network and their upstream target lncRNAs/circRNAs were selected and evaluated via quantitative real-time PCR (qPCR). According to the expression level in the two groups (FC value >2, *p* < 0.05) and the availability of probes (the probe sequences were queried via BLAST and compared with those of the rat genome), those specific to differentially expressed RNAs were subjected to qPCR. We extracted total RNA using an RNeasy Micro Kit (Qiagen, GmBH, Germany). Then, we pretreated and reverse transcribed the RNA into cDNA using a ReverTra Ace qPCR Kit (FSQ-101, Toyobo) and amplified the signal using a SYBR Green Kit (ABI, 4368708) on a QuantStudio 5 Real-Time PCR System (ABI, US). Reactions were performed following the manufacturer’s standard operating procedures. The primers used for mRNAs, circRNAs and lncRNAs were synthesized by Shanghai Biotechnology Corporation, and the sequences of these primers are listed in Table 1. PCR was performed in a 10 μl reaction mixture.

**TABLE 1:** Sequences of primers used for qRT-PCR analysis.

Gene symbol	Forward primer	Reverse primer
THBS2	CAG​GCC​CAA​GAC​TGG​CTA​CA	TGA​GTC​TGC​CAT​GAC​CTG​TTT​T
CHRM3	TCA​CTG​TTT​TGC​ATC​CTT​GTT​ACA	CAA​GGT​CAT​TGT​GAC​TCT​CTG​ACA​T
FPR2	TGCTGCTGGCAAGATGGA	TCA​TAG​GAC​ACT​TCT​TCA​TAT​TCA​TTC​A
BDKRB1	CCA​CCT​CAG​CCT​CTC​GAG​TT	CCAGGATGATGCCATGCA
CXCL1	GGC​ATA​CTG​CCT​TGT​TTA​ATG​GT	ACA​TCA​ATA​ATT​AAG​CCC​CTT​TGT​TC
ENST00000613838	TTC​ATC​TTG​GTG​GTC​GAG​TTT​CT	CTG​TCA​CTG​GTT​CCA​CCA​TGA​T
ENST00000645807	CGG​AAA​GAA​ACC​CCT​GGA​AT	TCC​TCA​GGT​TCC​TTG​CCA​TT
LINC01820:13	GAC​CCG​TCC​CAT​CCT​TAA​GAG	GTC​GAG​GAG​GTG​GGA​TCT​GA
ENST00000658492	TGC​AGG​GTG​GAC​ATA​GCT​CTA​A	GGT​CTC​TCA​TTT​CAC​ACA​GAA​CTT​G
ENST00000499452	AAG​CAG​TAA​TAA​AAG​TGG​GAA​GAC​CTA	TGA​GCC​CTC​GAG​AGA​AAA​TCA
GAPDH	TGA​CTT​CAA​CAG​CGA​CAC​CCA	CAC​CCT​GTT​GCT​GTA​GCC​AAA

### Statistical Analysis

We used the absolute number and frequency to describe the qualitative variables and used the mean and standard deviation to describe the quantitative variables. We used the Kolmogorov–Smirnov test to examine the normal distribution of all variables. Two-tailed Student’s t-tests were used to compare the discrepancy between the two groups, with a *p*-value ≤ 0.05 as the criterion of statistical significance. We used SPSS Statistics version 26.0 (IBM/SPSS, Inc., IL, United States) to proceed with all statistical analyses.

## Results

### mRNAs, circRNAs, lncRNAs and miRNAs Differentially Expressed Between the GO Group and the Normal Group

**FIGURE 1:**
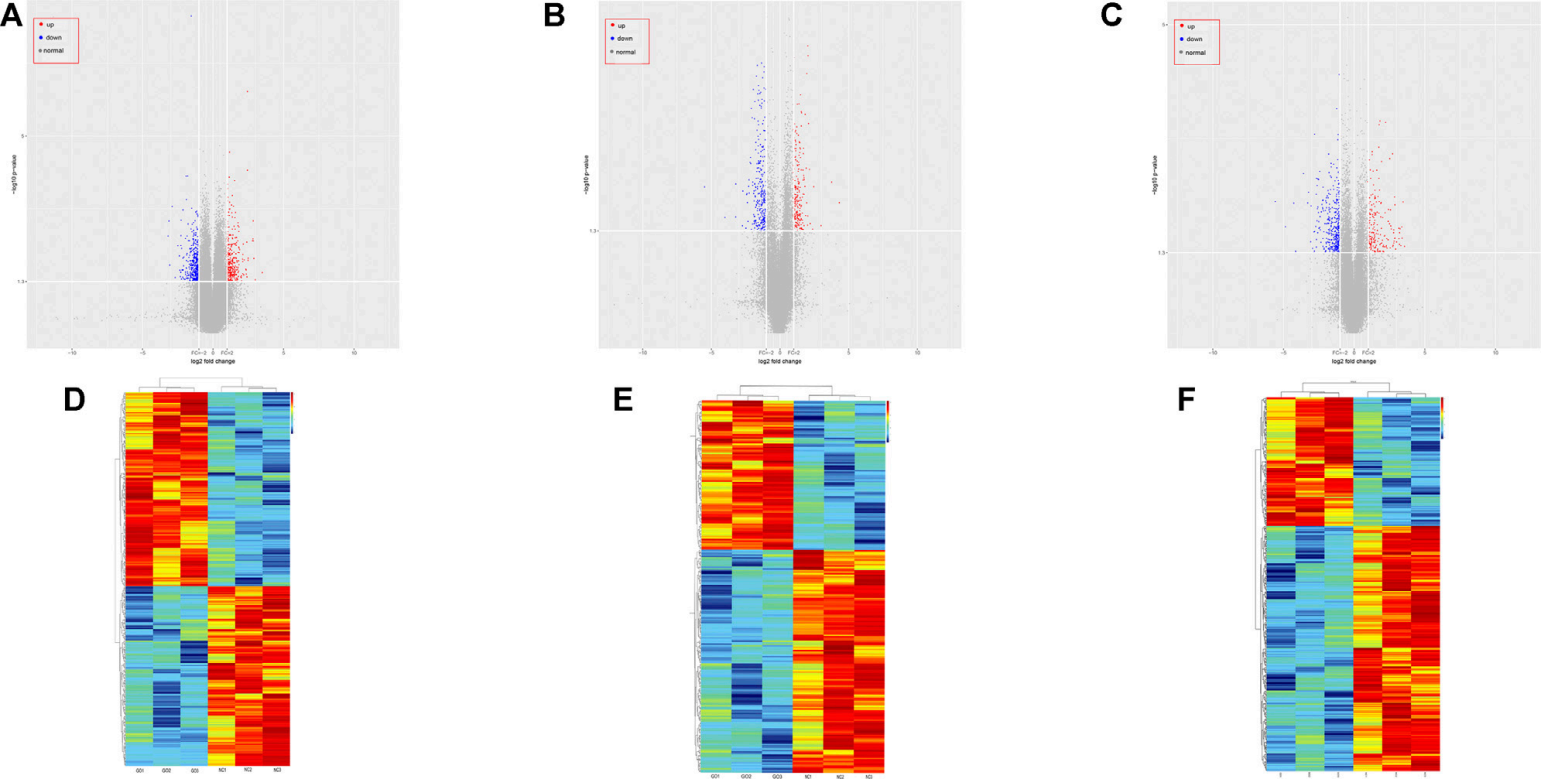
Overview of the expression profiles of ncRNA and mRNA sequencing data in paired Graves ophthalmopathy versus normal control groups. **(A)** Volcano plot of circRNA expression data for the paired GO group and control group. **(B)** Volcano plot of the lncRNA expression data of the same two groups. **(C)** Volcano plot of the mRNA expression data of the same two groups. **(D)** Hierarchical clustering heatmap of circRNA expression data from the same two groups. **(E)** Hierarchical clustering heatmap of the lncRNA expression data of the same two groups. **(F)** Hierarchical clustering heatmap of the mRNA expression data of the same two groups.

The raw and processed microarray analysis data have been deposited at GEO (https://www.ncbi.nlm.nih.gov/geo/query/acc.cgi?acc=GSE185952) and it also shown in Supplementary Tables. An overview of the expression of all selected RNAs in the GO group is shown in Figure 1 and Supplementary Figures S1–S3. Volcano plots were generated to visualize the differentially expressed RNAs between the two groups. In total, 361 mRNAs, 355 circRNAs, and 242 lncRNAs were differentially expressed between the GO group and the normal group according to the criteria of an FC > 2 and *p* < 0.05. Based on the differences in the RNA expression levels, hierarchical clustering revealed the profiles of RNAs that were differentially expressed for 3 pairs of GO and normal tissue samples.

### Establishment and Analysis of a PPI Network

**FIGURE 2:**
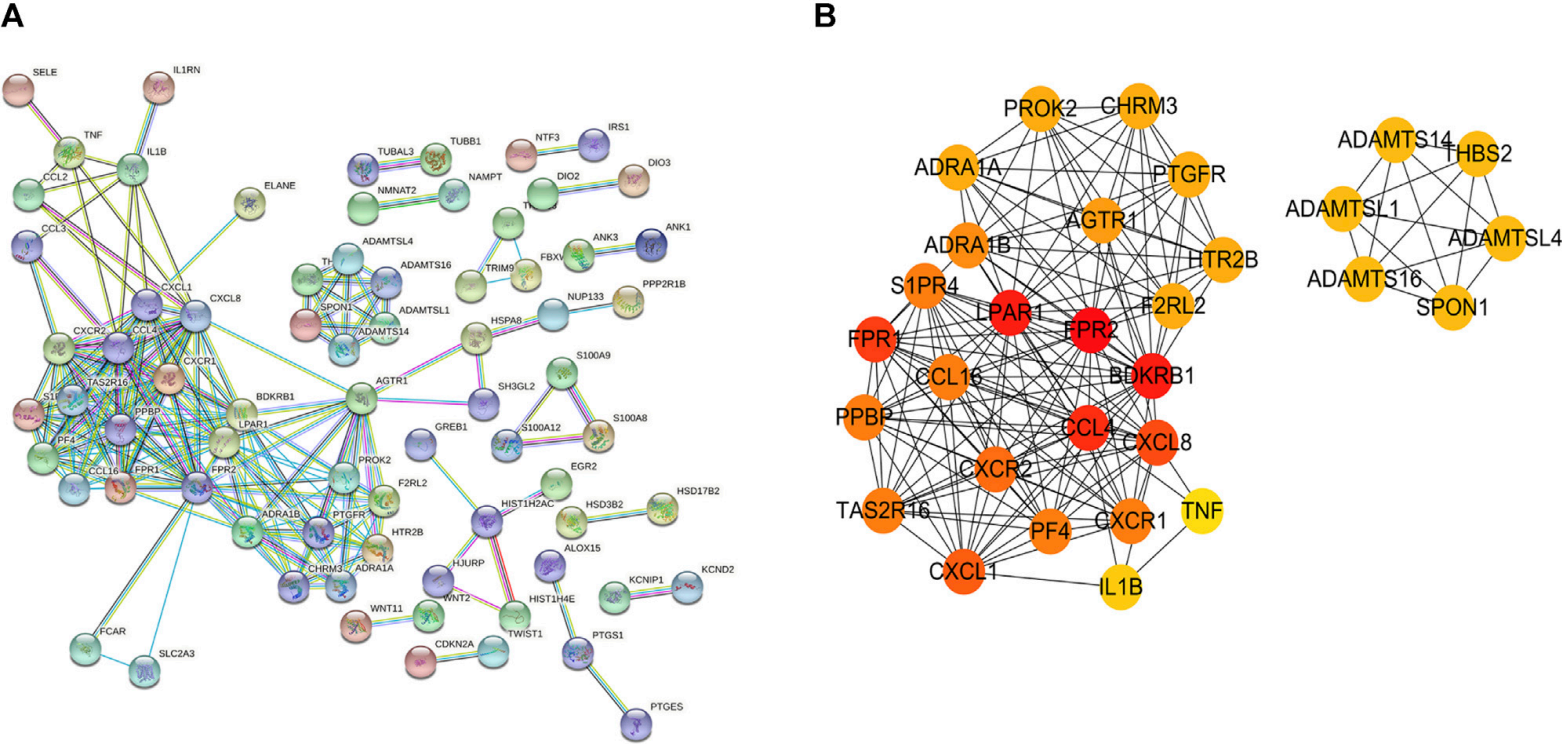
Top 30 hub genes identified in the PPI networks. **(A)** PPI network of differentially expressed genes. **(B)** Top 30 hub genes of differentially expressed mRNAs.

We constructed a PPI network of the differentially expressed mRNAs based on the data from our STRING analysis, and the results are shown in Figure 2A. According to node degree, several hub genes among these target genes were identified for visualization, and the interactors of the top 30 (Figure 2B) hub genes were reconstructed using Cytoscape software.

### Construction of the lncRNA/circRNA-miRNA-mRNA ceRNA Network for GO Analysis

**FIGURE 3:**
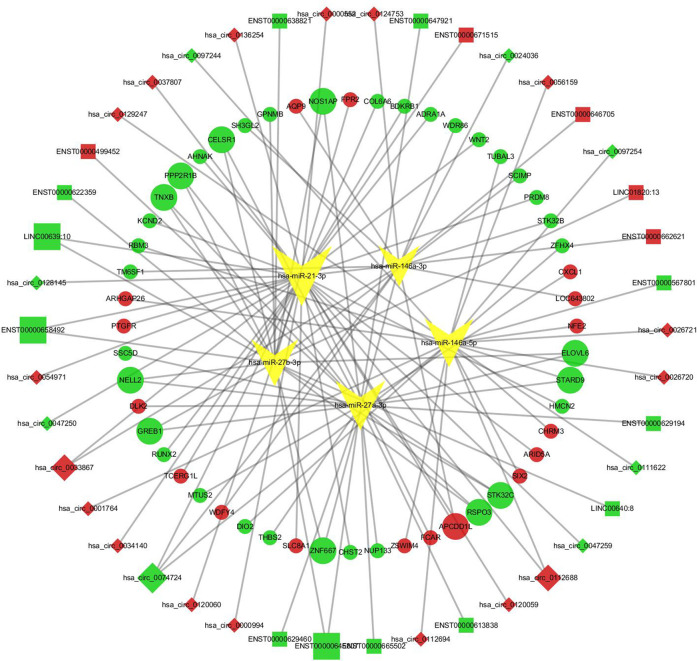
The lncRNA/circRNA-miRNA-mRNA ceRNA network. Squares represent lncRNAs and rhombus represent circRNAs, red means that RNAs were upregulated, green means downregulated, and yellow inverted triangles represent miRNAs. Round dots represent the mRNAs; similarly, red indicates that the RNAs were upregulated, and green indicates downregulated.

A total of 22144 miRNAs were found to be potentially related to the differentially expressed lncRNAs, 40626 miRNAs were found to potentially regulate the differentially expressed circRNAs, and 70307 miRNA were found to possibly regulate the differentially expressed mRNAs. Then, the lncRNA/circRNA-miRNA pairs that overlapped with the mRNA-miRNA pairs were taken as the intersecting pair. Five intersecting miRNAs of interest in the abovementioned pairs, miR-21, miR-27a, miR-27b, miR-146a and miR-155, were verified to have regulatory effects on the pathogenesis of GO (Kim et al., 2010; Li et al., 2014; Tong et al., 2015; Lee et al., 2016; Wang N. et al., 2017; Jang et al., 2019; Woeller et al., 2019). We used correlation coefficient analysis to intersect lncRNAs/circRNAs and mRNAs to find effective lncRNA-mRNA and circRNA-mRNA pairs. Finally, 166 pairs were found, and ceRNA networks were constructed (Figure 3).

### Bioinformatic Analyses

**FIGURE 4:**
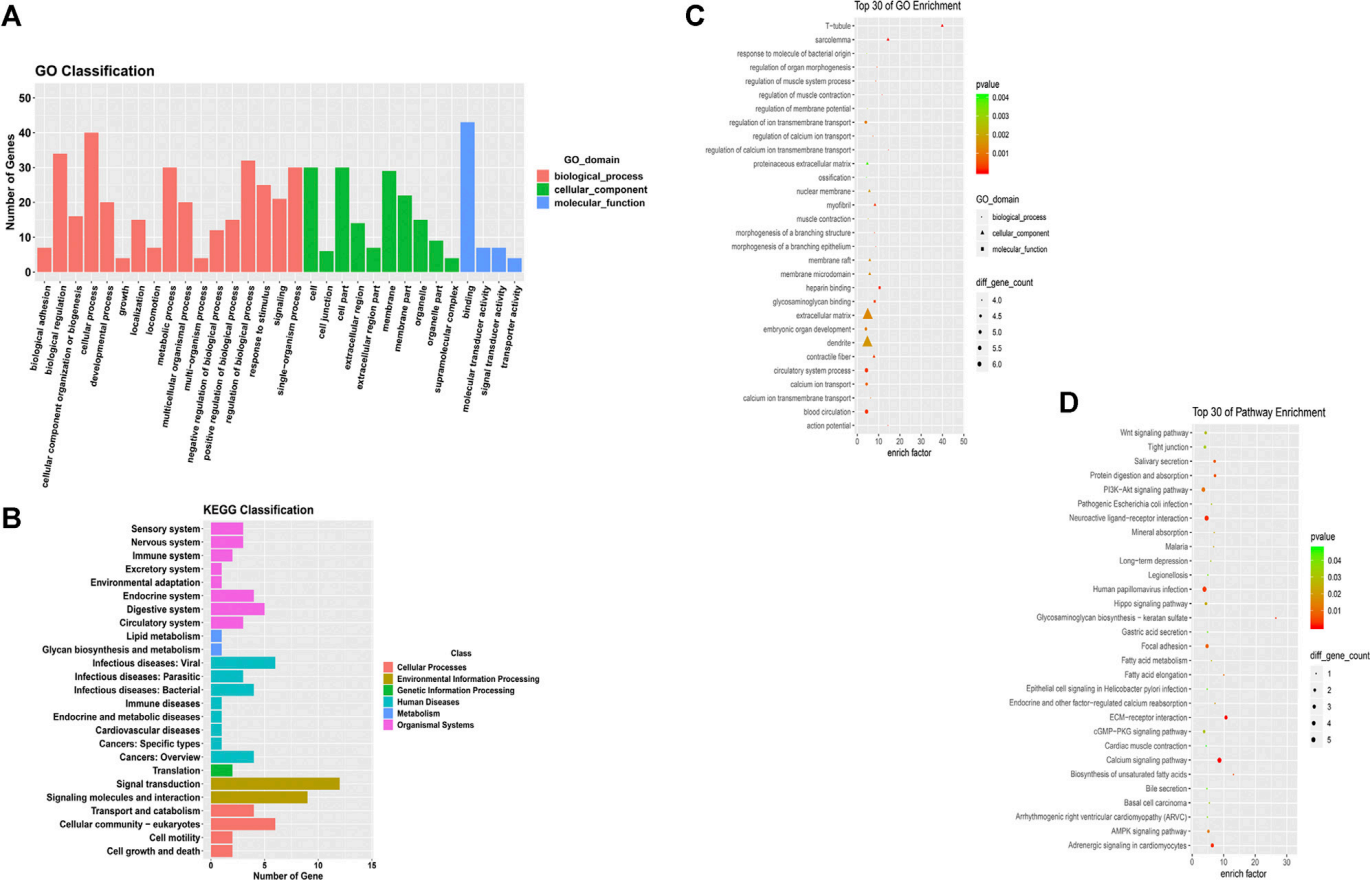
GO functional and pathway enrichment analyses of mRNAs in the co-expression network. (**A)** GO term classifications for differentially expressed mRNAs **(B)** Top 30 GO terms in the BP, CC and MF categories for differentially expressed mRNAs. **(C)** KEGG classifications for differentially expressed mRNAs **(D)** Top 30 enriched KEGG pathways for differentially expressed mRNAs.

We conducted Gene Ontology functional enrichment analysis and KEGG pathway analysis to predict the underlying biological effects and corresponding pathways of differentially expressed mRNAs from the ceRNA networks. Approximately 52 differentially expressed mRNAs verified by sequencing were identified in the ceRNA networks. The significantly enriched Gene Ontology terms of the differentially expressed mRNAs are shown in Figure 4 and the Gene Ontology classification of differentially expressed mRNAs are shown in Supplementary Figure S4. Based on the Gene Ontology functional enrichment analysis results, the differentially expressed mRNAs were mainly related to the BP categories biological regulation and cellular process, the CC category extracellular region, and the MF category signal transducer activity. Finally, pathway analysis of the differentially expressed mRNAs showed that multiple pathways were involved. Significant pathways of differentially expressed mRNAs are displayed in Figure 4 and the KEGG classification of differentially expressed mRNAs are shown in Supplementary Figure S5, suggesting that most of the differentially expressed mRNAs participate in the PI3K−Akt signaling pathway, extracellular matrix (ECM)-receptor interaction pathway and neuroactive ligand-receptor interaction pathway.

### Validation of Select Key RNAs

**FIGURE 5:**
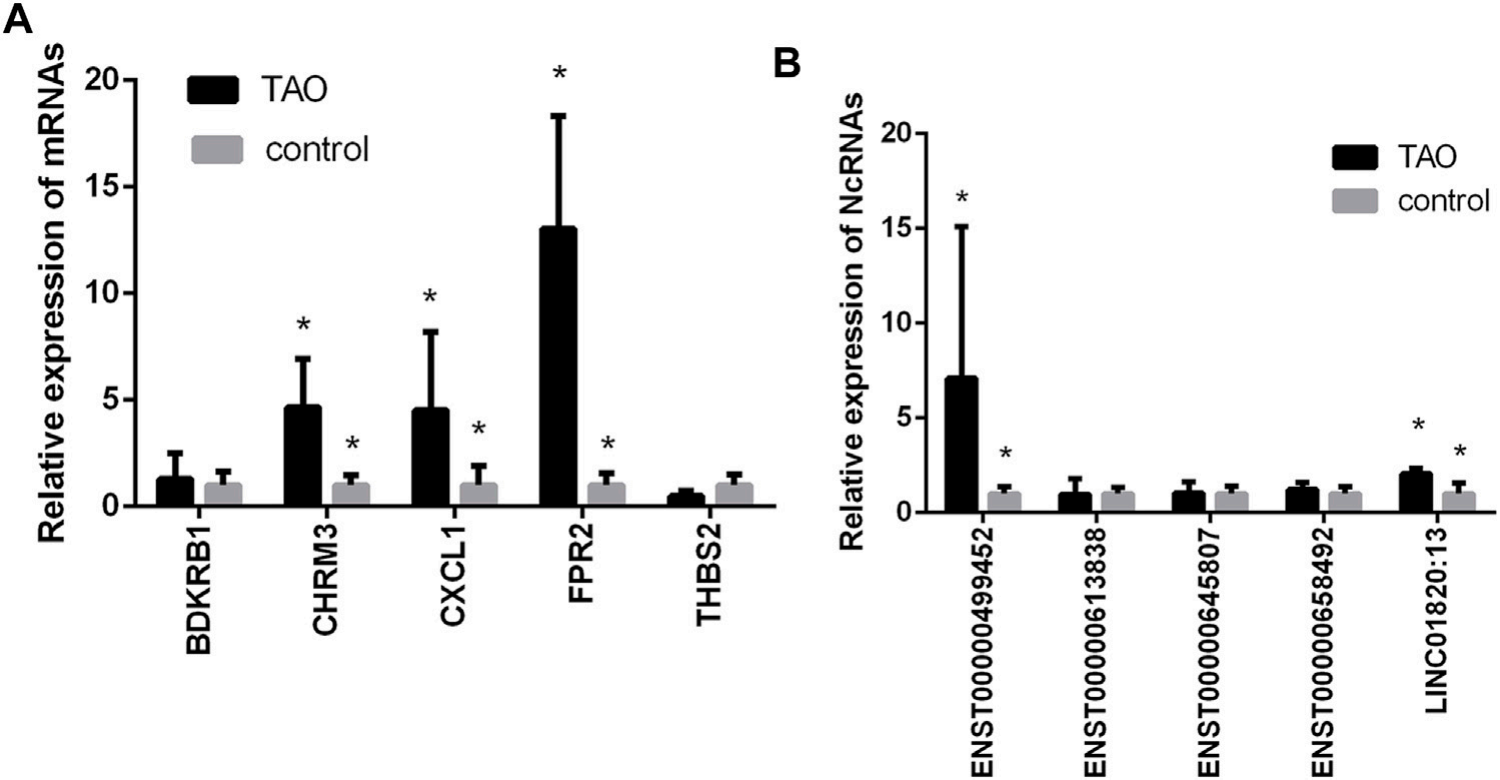
Validation of the expression levels of mRNAs and lncRNAs/circRNAs in the GO groups and control groups. **(A)** mRNA expression levels as verified by qPCR. **(B)** Expression levels of lncRNAs/circRNAs as verified by qPCR. (*, *p* < 0.05).

Based on the top 30 hub gene results and the abovementioned constructed ceRNA networks, we found that 5 mRNAs (THBS2, CHRM3, FPR2, BDKRB1, and CXCL1) not only were hub genes but also existed in the ceRNA network. These mRNAs and some of their upstream target lncRNAs/circRNAs (ENST00000613838, ENST00000645807, LINC01820:13, ENST00000658492, ENST00000499452) were subjected to qRT-PCR validation. The qRT-PCR results revealed that 4 mRNAs (THBS2, CHRM3, CXCL1, FPR2) and 2 lncRNAs (LINC01820:13, ENST00000499452) were differentially expressed between the GO group and the normal group (Figure 5).

## Discussion

GO is an autoimmune disease that frequently occurs in patients with Graves’ hyperthyroidism and occasionally occurs in euthyroid or hypothyroid patients. GO pathogenesis mainly involves autoimmune cross-reaction of the thyroid and extraocular muscles. However, the pathogenesis of GO has not been elucidated until now; thus, identifying specific biological indicators for early diagnosis and unambiguous classification methods for the severity and activity of GO are urgently needed. In addition, lncRNAs/circRNAs have been found to act critical characters in transcriptional interference and to be crucial adjectives for gene regulation, particularly in ceRNA networks due to the progress of high-throughput sequencing technologies (Liang et al., 2018; Zhang H. et al., 2019). Massive amounts of evidence have revealed that ceRNA-related genes greatly affect the occurrence process, development process and prognosis of most types of disease (Guttman and Rinn, 2012).

In our research, to better understand the molecular mechanisms of the ceRNA-related genes revealed by the GO analysis, we used RNA sequencing to recognize differentially expressed lncRNAs, circRNAs, and mRNAs by comparing healthy individuals with GO patients. After forecasting the lncRNA -miRNA interactions, circRNA -miRNA interactions and miRNA-mRNA interactions and conducting correlation coefficient analysis to identify effective lncRNA/circRNA-mRNA relationship pairs, we took the intersection of the two pairs and obtained the intersecting miRNAs. Then, 5 miRNAs of interest were verified as being involved in the pathogenesis of GO in previous research: miR-146a plays a negative regulatory role in the regulation of fibrosis in GO patients (Jang et al., 2018), and miR-155 is indispensable in the formation of fibrous tissue and mediates the TGF-β signaling pathway to drive collagen synthesis in GO patients (Li et al., 2014). miR-27b and miR-27a can inhibit adipogenesis in GO patients’ OFs (Jang et al., 2019), and miR-21 promotes fibrosis in OFs from GO patients (Tong et al., 2015). Furthermore, previous qPCR experiments showed that miR-146a, miR-155 and miR-21 are upregulated in the OFs of GO patients, whereas miR-27a and miR-27b are downregulated. A ceRNA regulatory network was subsequently constructed in association with the 5 miRNA relationship pairs, and Gene Ontology and KEGG pathway enrichment analyses of the mRNAs in the ceRNA network were performed. Among 361 mRNAs found to be differentially expressed between the GO group and the normal group, 52 were in the constructed ceRNA network. Gene Ontology analysis showed that the effects of mRNAs on TAO were mainly manifested in cell adhesion and the inflammatory response, probably because T cells infiltrate into the orbit through certain cell adhesion receptors, which initiate the autoimmune response of GO, and these cell adhesion receptors also act a costimulatory effect in T cell activation and promotion of antigen recognition (Heufelder, 1995). The Gene Ontology analysis results implied that these effects may be regulated at the transcriptional level. In addition, pathway enrichment analysis of the mRNAs showed that most potential mRNAs participated in the PI3K-Akt pathway and the ECM-receptor interaction pathway. Moreover, previous research showed that abnormal interactions between ECM proteins and T cells have indispensable effects on the pathogenesis of GO (Bednarczuk et al., 1998). ECM proteins are probably secondary autoantigens recognized by TAO antibodies and have the ability to bind to the ECM (Bednarczuk et al., 1999; Wu et al., 2020b). TSHR signaling induces cellular proliferation, proinflammatory cytokine production and adipogenesis in GO through the PI3K-Akt pathways, which are regulated by miR-146a and miR-155 (Ko et al., 2018; Woeller et al., 2019). Thus, we speculated that these effects might be regulated at the transcriptional level.

A novel strategy for constructing a lncRNA/circRNA-miRNA-mRNA ceRNA regulatory network was introduced based on the interactions among differentially expressed genes; the network comprised 17 lncRNAs, 25 circRNAs and 52 mRNAs. This comprehensive analysis network shrinks the scope of research and enhances the prediction accuracy for target lncRNAs/circRNAs with enormous potential to serve as therapeutic targets and candidate biomarkers for the diagnosis of GO patients. This ceRNA network includes numerous widely studied genes. For example, CXCL1 can induce angiotensin II and lead to myocardial hypertrophy, fibrosis and inflammation. In head and neck squamous cell carcinoma tissues, CXCL1 can regulate the activation of fibroblasts and upregulate proinflammatory factors (Wang D. et al., 2017; Wang L. et al., 2018). FPR2 plays an indispensable role in inflammation-related diseases and host immune regulation, and activation of its signaling pathway can lead to host immune response imbalance and inflammation. FPR2 is involved in a variety of diseases, including those caused by bacterial infection, inflammation, asthma, Alzheimer’s disease and cancer (Alessi et al., 2017; Sansbury et al., 2020). BDKRB1 is usually nonfunctional under state of physical health, yet it is rapidly induced after tissue damage, which can promote inflammation (Angers et al., 2005). The MFs of these mRNAs, such as regulation of the immune response and inflammatory response, seem to be related to the pathogenesis of GO, but their exact relationship with GO needs further research. Moreover, hsa_circ_0000552 and hsa_circ_00009914 were dysregulated in asthmatic airway epithelial cells and patients’ hip joint synovial tissues who underwent revision operations for aseptic loosening after total hip arthroplasty (Chen et al., 2021; Ni et al., 2021), which probably indicates that they have potential effects on these two diseases. Moreover, the constructed ceRNA network identified not only a series of RNAs with verified specific functions but also potentially unexplored RNAs, such as LINC01820:13, which showed high positive correlations with FPR2 and hsa-miR-27b-3p, and ENST00000499452, which is related to CXCL1 and hsa-miR-27a-3p.

Based on the qPCR results, two ceRNA pathways were verified to be dysregulated throughout the whole network and to have potential effects on GO patients: the LINC01820:13-hsa-miR-27b-3p-FPR2 pathway and ENST00000499452-hsa-miR-27a-3p-CXCL1 pathway. The biological functions of FPR2, CXCL1, hsa-miR-27b-3p, and hsa-miR-27a-3p are described above. Based on the regulatory function of FPR2 and miR-27b and the mechanism of ceRNA, we speculate that LINC01820:13 reduces the inhibitory effect of miR-27b on FPR2 by competitively binding to the RISC of miR-27b, thereby upregulating the expression of FPR2 and leading to an autoimmune response and inflammation in GO patients. ENST00000499452 competitively inhibits miR-27a through the same mechanism, accordingly weakening its inhibitory effect on CXCL1 and strengthening the immune response and inflammatory response and even fibrosis in OFs in TAO patients. However, the exact role of these RNAs in the pathogenesis of GO and whether there is a clear targeting effect between LINC01820:13, hsa-miR-27b-3p and FPR2 and between ENST00000499452, hsa-miR-27a-3p and CXCL1 still need to be further explored.

There are several limitations of this research. First, OFs from GO patients at the active stage were not included in our experiment. The first-line treatment for GO at the active stage is methylprednisolone pulse therapy before orbital decompression. Because RNA expression is probably influenced by dexamethasone, it is difficult to obtain OFs from glucocorticoid-free patients with active GO. Second, the number of cases used for sequencing was small, and the representative intensity was not large. Limited by the experimental conditions, we performed qPCR verification on only a few genes of interest and did not include all the RNAs in the ceRNA network. Third, a recognized animal model of GO is still lacking, and we plan to bring animal experiments into our further research. Finally, the bioinformatic analysis in our research is relatively thin, and we plan to introduce more analyses in our future research. Despite these limitations, our research is the first to attempt to construct a novel lncRNA/circRNA-miRNA-mRNA ceRNA network in GO to provide innovative ideas and potential genes for research by other authors to identify GO biomarkers and potential therapeutic targets.

In conclusion, we constructed an innovative lncRNA/circRNA-miRNA-mRNA ceRNA network in GO and identified a large number of potential therapeutic targets and candidate biomarkers for diagnosis in GO patients. Furthermore, we found two significant pathways, the LINC01820:13-hsa-miR-27b-3p-FPR2 ceRNA pathway and the ENST00000499452-hsa-miR-27a-3p-CXCL1 pathway, which probably affect the autoimmune response and inflammation in GO patients. However, our team and members of other laboratories should use this network and conduct more studies to further validate these findings.

## Data Availability

The original contributions presented in the study are publicly available in NCBI under accession number GSE185952.
