# Supplementary material for: A Novel Competing Endogenous RNA Network Associated With the Pathogenesis of Graves’ Ophthalmopathy
Source: Front Genet. 2021 Dec 15;12:795546. doi: 10.3389/fgene.2021.795546 (PMC8714659; doi:10.3389/fgene.2021.795546)
Supplement: Supplementary file 3 [file Table1.DOCX]

Supplementary Material

# Supplementary Figures and Tables

## Supplementary Figures

**Supplementary Figure 1.** Scatter plot of circRNA expression data for the paired GO group and control group. Round dots represent circRNAs, red means that RNAs were upregulated, blue means downregulated,and gray means RNAs were normal expressed.

**Supplementary Figure 2.** Scatter plot of lncRNA expression data for the paired GO group and control group. Round dots represent lncRNAs, red means that RNAs were upregulated, blue means downregulated,and gray means RNAs were normal expressed.

**Supplementary Figure 3.** Scatter plot of mRNA expression data for the paired GO group and control group. Round dots represent mRNAs, red means that RNAs were upregulated, blue means downregulated,and gray means RNAs were normal expressed.

**Supplementary Figure 4.** GO classification of differentially expressed mRNAs from the ceRNA networks.

**Supplementary Figure 5.** KEGG classification of differentially expressed mRNAs from the ceRNA networks.

## Supplementary Tables

**Supplementary Table 1.**Differentially expressed genes. The differentially expressed genes were screened by fold-change and Student's T-test.The sheet 1 are differentially expressed circRNAs,the sheet 2 are differentially expressed lncRNAs,the sheet 3 are differentially expressed mRNAs.

**Supplementary Table 2.**CircRNA target genes. By analyzing the possible binding sites of circRNA and miRNA, the miRNA molecules adsorbed by circRNA were predicted.

**Supplementary Table 3.**LncRNA target genes. By analyzing the possible binding sites of lncRNA and miRNA, the miRNA molecules adsorbed by lncRNA were predicted.The sheet1 used Cis regulates target gene prediction methods,and the sheet 2 used Trans regulatory target gene prediction method.

**Supplementary Table 4.**CeRNA network RNA pairs.This table contains all of the RNA pairs involve in the constructed ceRNA network.

**Supplementary Table 5.** GO functional enrichment analyses results of differentially expressed mRNAs from the ceRNA networks.

**Supplementary Table 6.** KEGG enrichment analyses results of differentially expressed mRNAs from the ceRNA networks.

**Supplementary Table 7.**qRT-PCR results. Ct value comparison method was used for data analysis,and results were shown in the table.
