# Supplementary figures and images for: A Novel Competing Endogenous RNA Network Associated With the Pathogenesis of Graves’ Ophthalmopathy
Source: Front Genet. 2021 Dec 15;12:795546. doi: 10.3389/fgene.2021.795546 (PMC8714659; doi:10.3389/fgene.2021.795546)

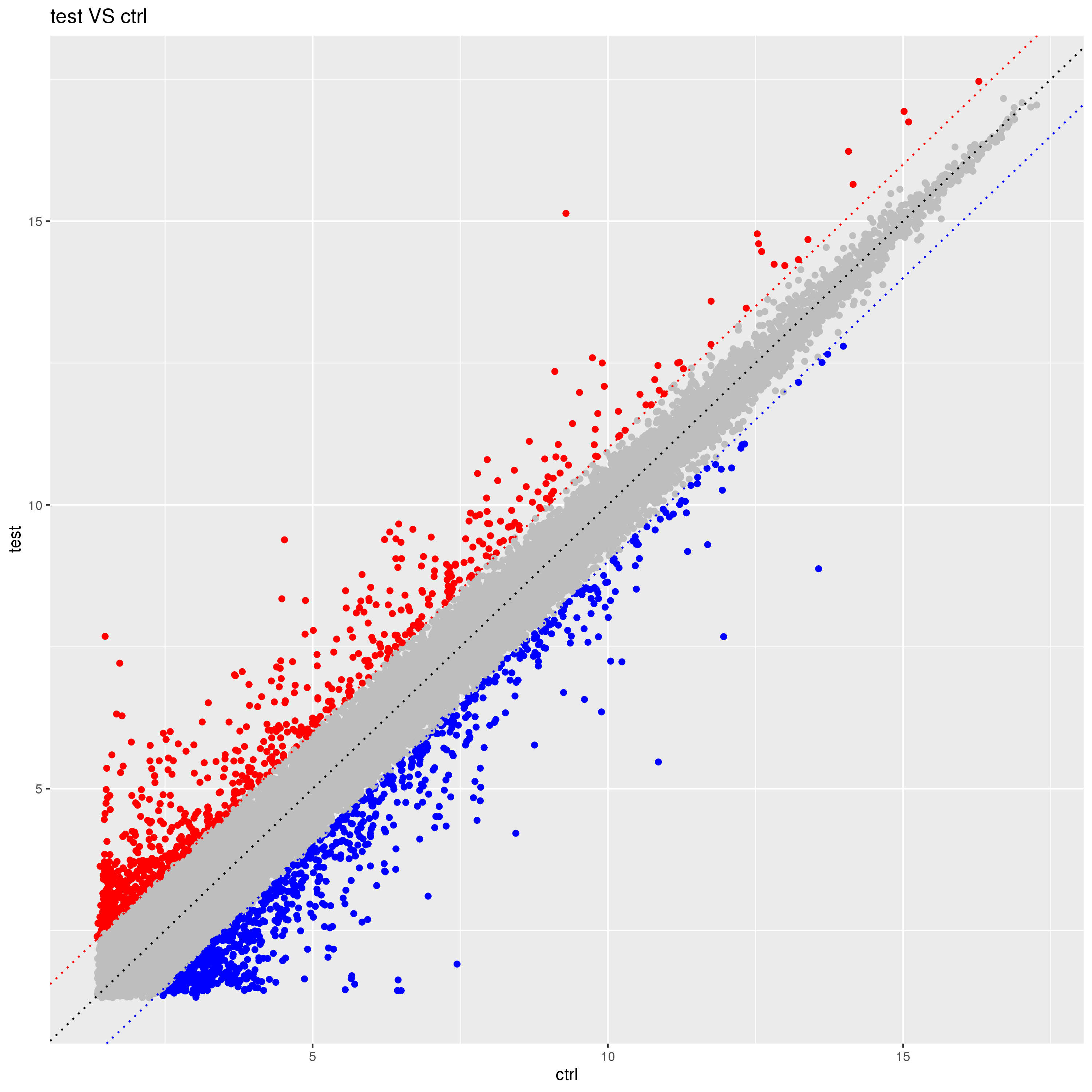

Supplement: Supplementary file 4 [file Image3.TIF]

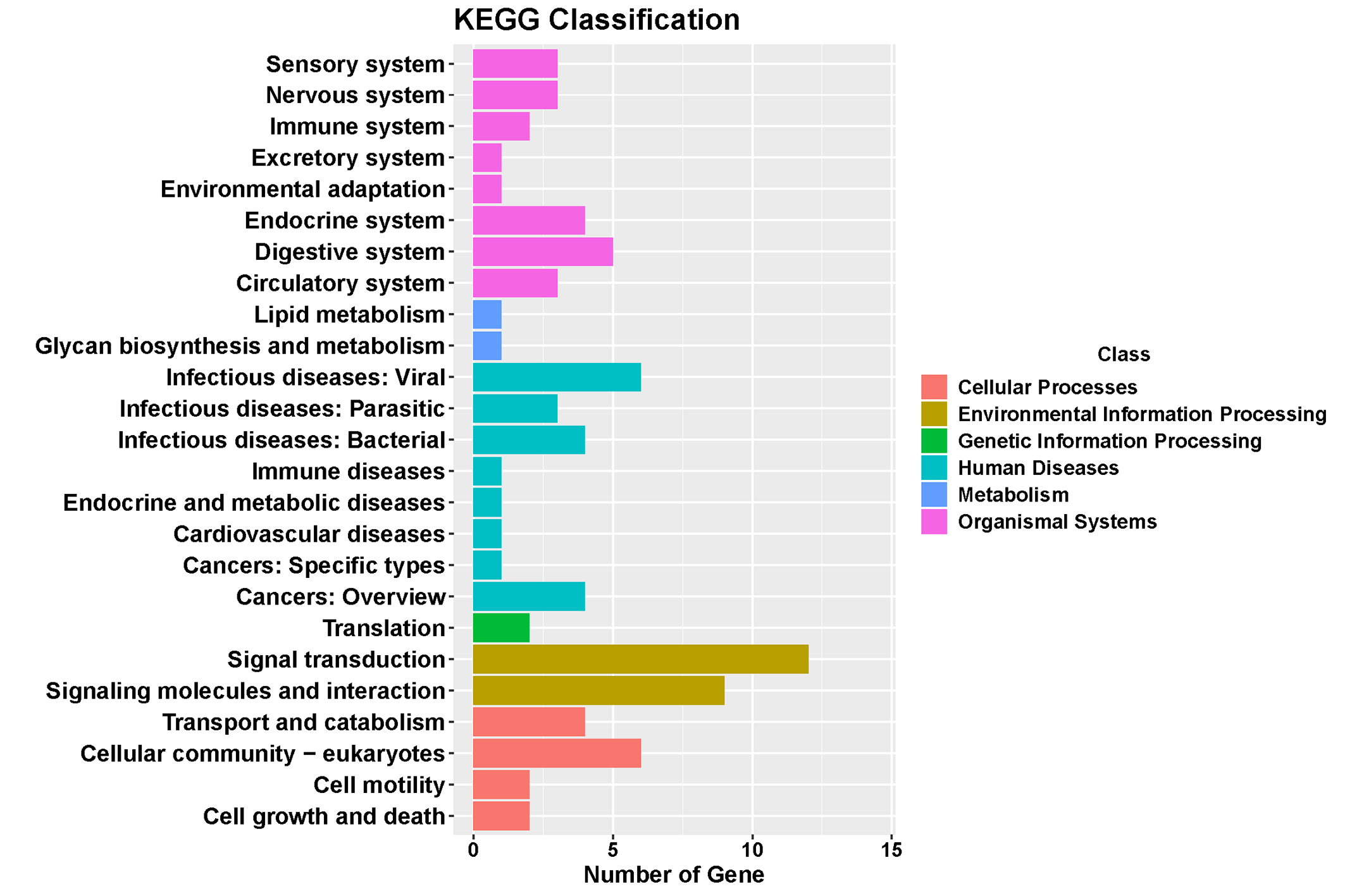

Supplement: Supplementary file 5 [file Image5.TIFF]

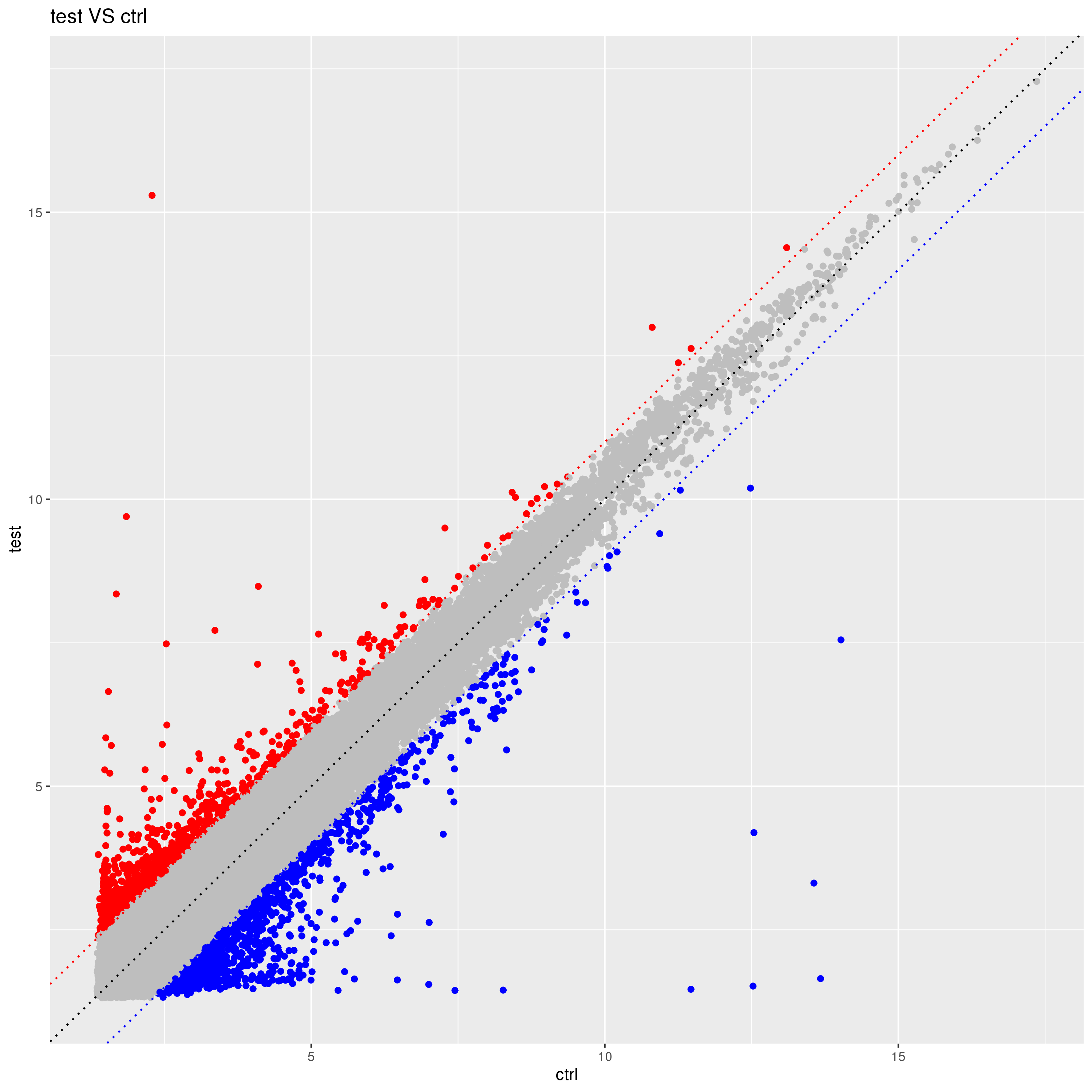

Supplement: Supplementary file 6 [file Image2.TIF]

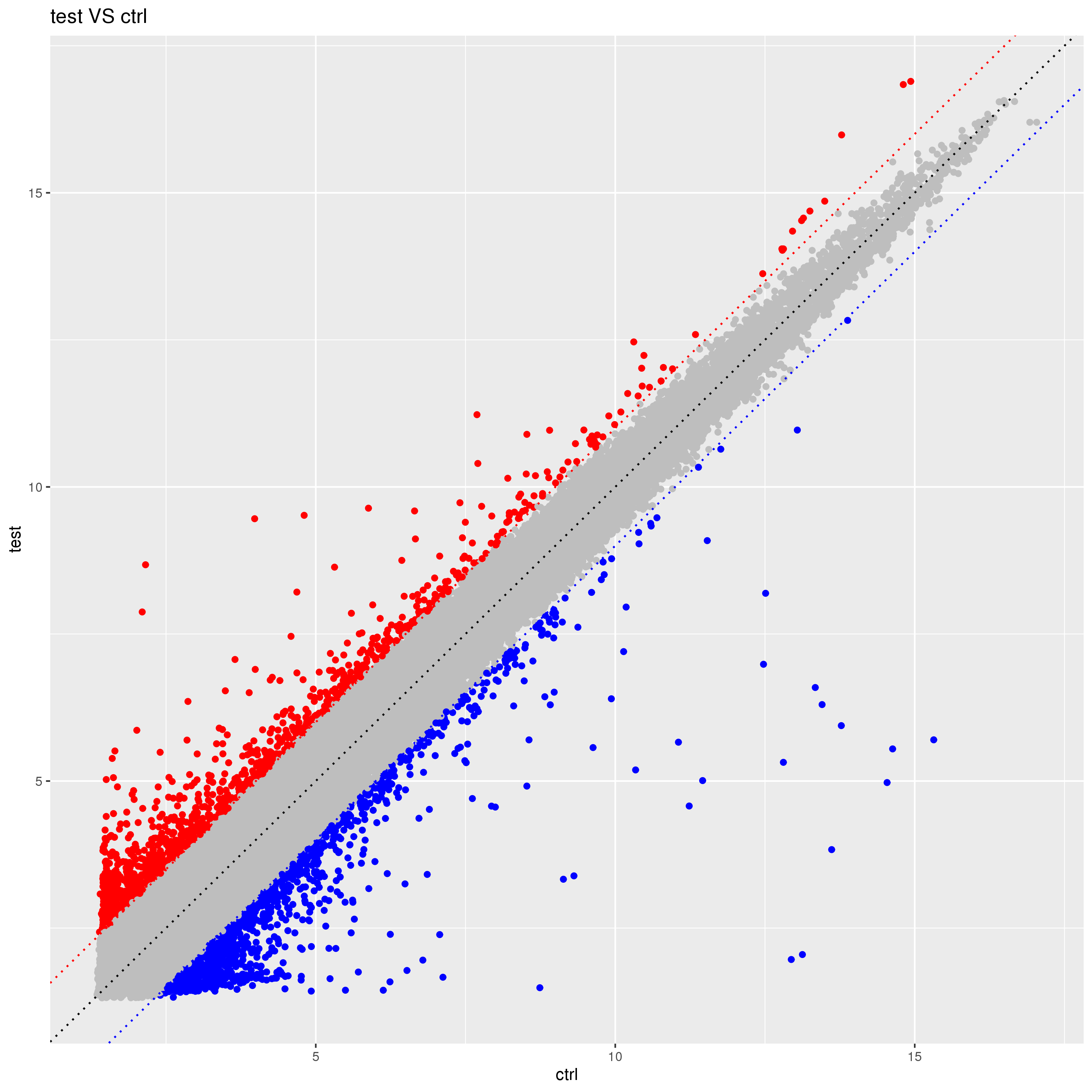

Supplement: Supplementary file 7 [file Image1.TIF]

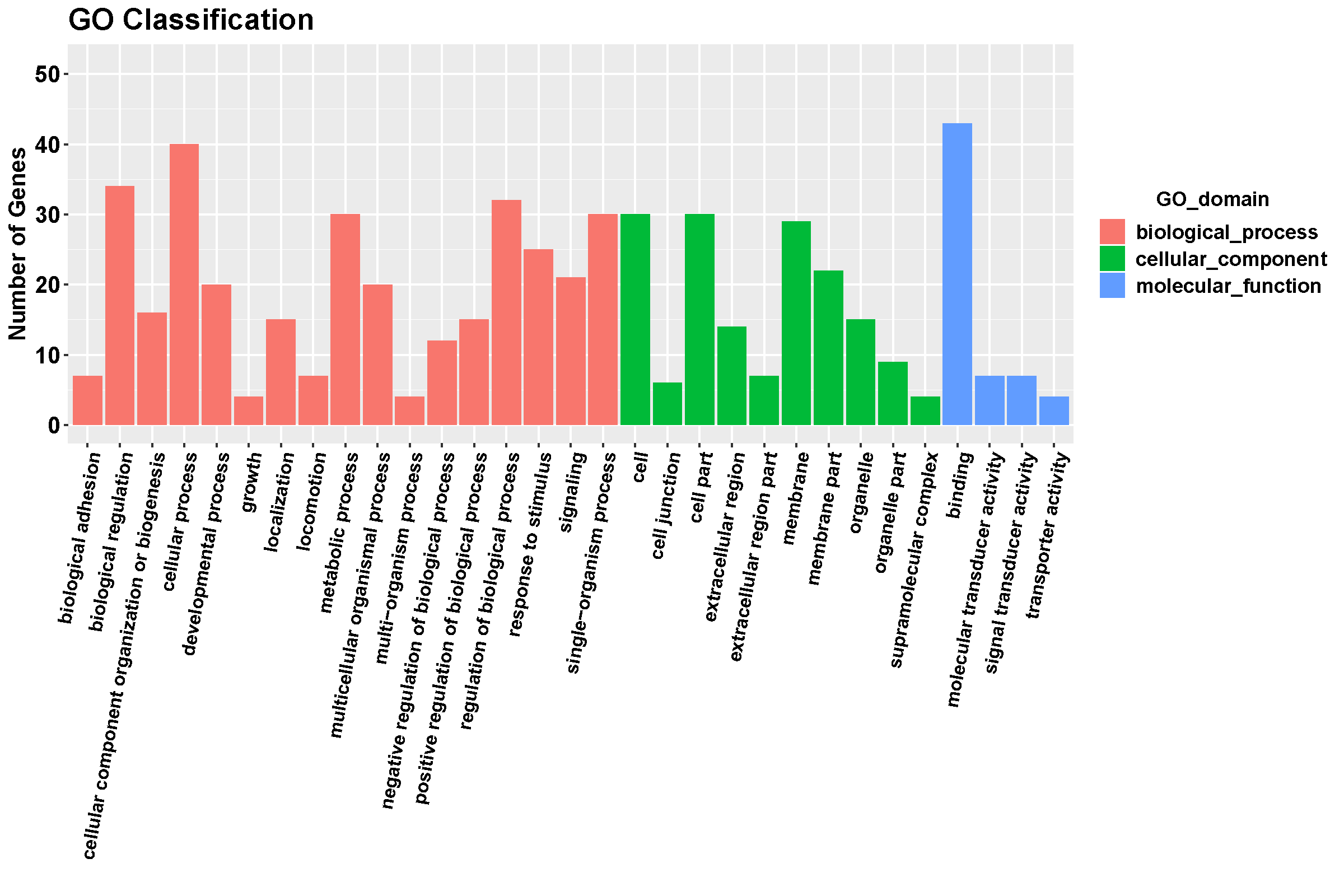

Supplement: Supplementary file 11 [file Image4.TIFF]
